# Clustered Regularly Interspaced short palindromic repeats‐Based Microfluidic System in Infectious Diseases Diagnosis: Current Status, Challenges, and Perspectives

**DOI:** 10.1002/advs.202204172

**Published:** 2022-10-18

**Authors:** Yi Xie, Huimin Li, Fumin Chen, Srisruthi Udayakumar, Khyati Arora, Hui Chen, Yang Lan, Qinqin Hu, Xiaonong Zhou, Xiaokui Guo, Leshan Xiu, Kun Yin

**Affiliations:** ^1^ School of Global Health Chinese Center for Tropical Diseases Research Shanghai Jiao Tong University School of Medicine Shanghai 200025 P. R. China; ^2^ One Health Center Shanghai Jiao Tong University‐The University of Edinburgh Shanghai 200025 P. R. China; ^3^ Division of Engineering in Medicine Department of Medicine Brigham and Women's Hospital and Harvard Medical School Boston MA 02139 USA; ^4^ Centre for Nature‐Inspired Engineering Department of Chemical Engineering University College London London WC1E 7JE UK

**Keywords:** clustered regularly interspaced short palindromic repeats/clustered regularly interspaced short palindromic repeats‐associated proteins biosensing mechanisms, infectious diseases diagnosis, integrated detection, microfluidic platforms

## Abstract

Mitigating the spread of global infectious diseases requires rapid and accurate diagnostic tools. Conventional diagnostic techniques for infectious diseases typically require sophisticated equipment and are time consuming. Emerging clustered regularly interspaced short palindromic repeats (CRISPR)/CRISPR‐associated proteins (Cas) detection systems have shown remarkable potential as next‐generation diagnostic tools to achieve rapid, sensitive, specific, and field‐deployable diagnoses of infectious diseases, based on state‐of‐the‐art microfluidic platforms. Therefore, a review of recent advances in CRISPR‐based microfluidic systems for infectious diseases diagnosis is urgently required. This review highlights the mechanisms of CRISPR/Cas biosensing and cutting‐edge microfluidic devices including paper, digital, and integrated wearable platforms. Strategies to simplify sample pretreatment, improve diagnostic performance, and achieve integrated detection are discussed. Current challenges and future perspectives contributing to the development of more effective CRISPR‐based microfluidic diagnostic systems are also proposed.

## Graphical abstract

The CRISPR/Cas detection systems that are armed with state‐of‐the‐art microfluidic platforms have shown remarkable potential as next‐generation diagnostic tools. In this review, CRISPR‐based microfluidic systems for infectious diseases diagnosis, including the mechanisms of CRISPR/Cas biosensing and the cutting‐edge microfluidic devices are systematically summarized. Additionally, current challenges and future perspectives are discussed to develop more effective CRISPR‐based microfluidic diagnostic systems.

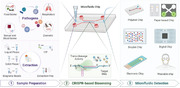

## 1 Introduction

Infectious diseases caused by pathogenic microorganisms have been the leading cause of human morbidity and mortality, despite considerable advancements in diagnosis and treatment.^[^ ^1^ ^]^ Pathogens may be spread through respiratory tract infections or blood‐borne, sexual, and fecal‐oral transmission.^[^ ^2^ ^]^ Recent outbreaks of infectious diseases such as acquired immunodeficiency syndrome, Zika, Ebola, and coronavirus disease 2019 (COVID‐19) have increased the global health burden.^[^ ^3^, ^4^ ^]^ The COVID‐19 pandemic, caused by the severe acute respiratory syndrome coronavirus 2 (SARS‐CoV‐2), has rapidly spread worldwide as a public health emergency. The World Health Organization has reported that the loss of lives of millions of people because of the lack of effective therapeutics poses a serious threat to regional stability and prosperity.^[^ ^5^ ^]^

Mitigating the spread of infectious diseases requires practical diagnostic tools that are user‐friendly, rapid, sensitive, specific, and field‐deployable.^[^ ^6^ ^]^ Molecular diagnostic methods, including polymerase chain reaction (PCR) and next‐generation sequencing (NGS), have exhibited excellent sensitivity and specificity. However, these diagnostic tools are generally used in centralized laboratories and requires specialized equipment, which is cumbersome, costly, and unsuitable in resource‐limited settings.^[^ ^7^, ^8^ ^]^ Recently, the clustered regularly interspaced short palindromic repeats (CRISPR)/CRISPR‐associated proteins (Cas) system has attracted considerable scientific interest. In addition to its application in gene editing and cancer therapy, CRISPR/Cas technology has also been used in molecular diagnosis, involving the selective activation of the nuclease activity of Cas‐associated proteins under the direction of a guide RNA to target nucleic acids.^[^ ^9^, ^10^, ^11^ ^]^ For example, groundbreaking CRISPR/Cas biosensing methods, including SHERLOCK (specific high‐sensitivity enzymatic reporter unlocking) and DETECTR (DNA endonuclease‐targeted CRISPR trans reporter), have successfully achieved sensitive pathogen detection and genotyping.^[^ ^12^ ^]^ Although CRISPR/Cas systems have the merits of modularity, programmability, and high specificity, they are limited in point‐of‐care testing (POCT) and lack portable diagnostic platforms.^[^ ^13^, ^14^, ^15^ ^]^ As field‐deployable detection is needed for infectious diseases diagnosis and surveillance, epidemiological studies, and fundamental research, it is essential to combine the CRISPR/Cas system into integrated devices for automatic, high‐throughput, and in situ diagnosis.^[^ ^16^ ^]^

Microfluidic platforms promise exciting solutions for field‐deployable detection as they integrate operation, precise control, and fluidic manipulation in a microscale environment.^[^ ^17^ ^]^ Automated microfluidic biosensing facilitates miniaturized and integrated diagnosis, which was previously completed by separate processes with enhanced speed and efficiency. Meanwhile, the simple design and fabrication of microfluidic devices enhances their accessibility to less‐resourced settings, accelerating the generation of various microfluidic detection methods for infectious diseases.^[^ ^18^, ^19^ ^]^ By exploiting the advantages of microfluidics, the next‐generation molecular diagnosis technique can be refined with an accurate CRISPR/Cas system, which has significant advantages, including lower sample consumption, cost‐effectiveness, short turnaround time, high specificity, and the possibility of integrating multiplex testing.^[^ ^20^, ^21^, ^22^ ^]^ To highlight this emerging innovation, it is urgent and necessary to summarize recent advances in CRISPR‐based microfluidic systems for infectious diseases diagnosis.

**Figure 1:**
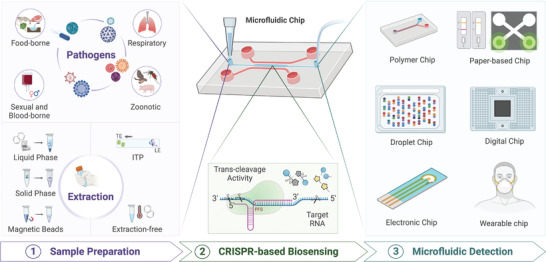
Overview of CRISPR‐based microfluidic system in infectious diseases diagnosis.

This review summarizes the latest advances in CRISPR‐based microfluidic systems for the diagnosis of infectious diseases (**Figure** **1**). We review sample pretreatment methods and the mechanisms and applications of various CRISPR/Cas systems in bioanalysis. Critical aspects of recent advancements toward the development of CRISPR‐based microfluidic platforms, including cutting‐edge paper‐based, digital detection, and autonomous wearable diagnosis, have also been described. This review presents a snapshot of the promising technological advances in this field and discusses the current challenges and future perspectives, which will contribute to the development of more efficient biosensing strategies and effective CRISPR‐based microfluidic techniques for infectious diseases diagnosis.

## 2 Sample Preparation

**Figure 2:**
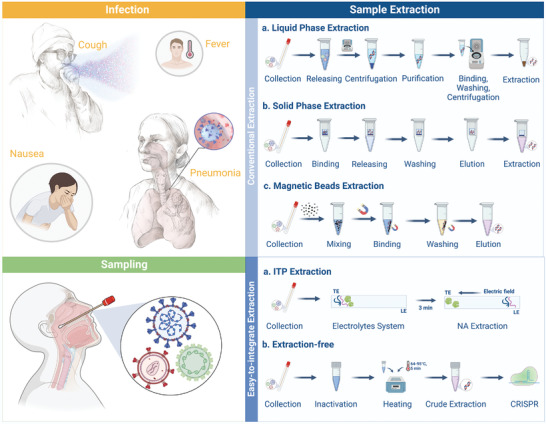
Workflow of sample preparation in CRISPR‐based diagnosis.

Sample preparation with efficient pathogen concentration and extraction and purification of target analytes is a crucial prerequisite for integrated CRISPR‐based microfluidic platforms with deployable field capabilities. Efficient CRISPR/Cas diagnosis can be achieved by removing interfering molecules from raw samples, including blood, urine, or saliva. Typically, sample preparation requires complicated sequential steps that may differ significantly between sample types or target analytes. For example, malaria diagnosis relies on collecting parasite‐infected red blood cells (RBCs); however, RBCs usually need to be removed for pathogen concentration.^[^ ^23^ ^]^ Therefore, pathogen enrichment and matrix removal are essential for effectively detecting CRISPR‐based microfluidic platforms, particularly for clinical or environmental samples with low pathogen concentrations and complex constituents.^[^ ^24^, ^25^ ^]^ Although conventional sample pretreatment is implemented in the centralized laboratory, the microfluidic technique represents a simplified alternative, such as employing a filtration step without a centrifuge. Cell lysis to release target analytes is a crucial step that can be integrated into microfluidic devices through thermal, chemical, enzymatic, mechanical, and electrical treatments, according to the acquired quality of extraction and sample type. Herein, we emphasize the paramount role of reliable and accessible extraction methods (**Figure** **2**), which are particularly important when considering the demand for innovations in future CRISPR‐based POC strategies for various diagnostic purposes.

### 2.1 Conventional Extraction

To achieve rapid preparation of raw samples, several conventional nucleic acid extraction strategies, such as liquid‐phase extraction, solid‐phase extraction, and magnetic bead extraction, have been established, but are usually performed in well‐equipped laboratories by skilled technicians. Among these methods, liquid‐phase extraction is a widely used technique for nucleic acid purification owing to its relative solubility.^[^ ^26^ ^]^ Convenient liquid extraction has been used in microfluidic devices with integrated liquid‐phase sample preparation components.^[^ ^27^ ^]^ By illustration, Qin et al. developed an automated microfluidic chip that integrated RNA extraction, amplification, and CRISPR‐based detection. RNA extraction was performed by chloroform incubation, centrifugation, and an ethanol wash.^[^ ^28^ ^]^ Although this widely adopted approach is compelling in terms of its inexpensiveness and high extraction efficiency, the sample processing step is time‐consuming and hazardous to the environment, requiring trained laboratory personnel and specialized instruments.^[^ ^29^, ^30^, ^31^ ^]^

In addition to liquid‐phase extraction, solid‐phase extraction utilizes a tangible medium (such as micropillars or membranes) to extract nucleic acids by combining the dehydration of nucleic acid molecules, hydrogen bonding, and electrostatic interactions. Compared to liquid‐phase extraction, this flexible and reversible affinity procedure should increase nucleic acid binding efficiency without introducing hazardous chemicals.^[^ ^32^, ^33^ ^]^ For example, commercial silica‐based membrane extraction kits are often used for CRISPR‐based detection of pathogens because of their efficiency, rapidity, and simplicity.^[^ ^13^, ^18^, ^34^, ^35^, ^36^, ^37^, ^38^, ^39^, ^40^, ^41^ ^]^ Although this strategy is effective, solid‐phase extraction methods have significant drawbacks, including iterative sample‐handling steps, limiting their integration into microfluidic devices. With the all‐in‐one microfluidic platform developed for detecting nucleic acid biomarkers by combining solid‐phase extraction, nucleic acid amplification, and detection processes, solid‐phase extraction has the potential to be integrated into microfluidic systems.^[^ ^42^ ^]^

The conventional magnetic bead extraction method directly mixes samples with magnetic beads and automatically moves the beads with an artificial external magnetic field to achieve nucleic acid purification.^[^ ^43^ ^]^ To date, magnetic bead extraction has been used in several CRISPR‐based microfluidic systems.^[^ ^12^, ^20^, ^22^, ^44^, ^45^, ^46^ ^]^ Notably, Chen et al. refined this method by designing a droplet magnetofluidic (DM) device for sample preparation, which was integrated into a one‐step CRISPR/Cas‐assisted assay for the automated point‐of‐care detection of SARS‐CoV‐2.^[^ ^47^ ^]^ Once the samples were injected into an assay cartridge and placed in the DM device, automatic sample preparation with nucleic acid purification using magnetic beads, CRISPR‐based reaction process, and fluorescence output successfully occurred in this palm‐sized POCT device. However, effective CRISPR‐based microfluidic diagnosis still requires simple sample pretreatment with a minimal hand‐operated process, which has driven the search for alternative nucleic acid extraction techniques.

### 2.2 Easy‐to‐Integrate Extraction

Easy‐to‐integrate sample preparation is ideal for CRISPR‐based microfluidic diagnostics, which involves all steps from raw sample processing to signal detection and is autonomous with minimal human intervention. Isotachophoresis (ITP) is a nucleic acid isolation technique applied in a discontinuous electrolyte system, where the target analytes are separated based on the differences in their mobility upon application of pressure to an electric field.^[^ ^48^, ^49^ ^]^ Compared to the liquid/solid phase extraction methods, which fail to extract DNA present at concentrations less than 0.1 ng and 1 ng, respectively, the ITP method can be implemented with small sample volumes and automated to obtain purified and amplifiable nucleic acids.^[^ ^50^, ^51^, ^52^, ^53^ ^]^ In addition, ITP is compatible with amplification and the CRISPR/Cas reaction, which can be easily incorporated into microfluidic devices.^[^ ^54^, ^55^, ^56^ ^]^ Ramachandran et al. utilized this automated method to purify RNA from raw samples in combination with loop‐mediated isothermal amplification (LAMP) and an ITP‐enhanced CRISPR/Cas assay to detect SARS‐CoV‐2 RNA in a microfluidic chip within 35 min.^[^ ^57^ ^]^ Therefore, the ITP method shows excellent potential for use in miniaturized and integrated CRISPR‐based microfluidic devices designed for the rapid and field‐deployable detection of infectious diseases from raw samples.

Extraction‐free methods that do not require the application of lysis reagents that may be incompatible with CRISPR/Cas detection are ideal candidates to meet the requirements for feasibility of microfluidic devices.^[^ ^58^, ^59^, ^60^ ^]^ The extraction‐free approach has excellent advantages, including high tolerance to potential inhibitors, time savings, and ease of optimization.^[^ ^61^, ^62^, ^63^, ^64^ ^]^ For example, Azmi et al. developed CASSPIT (Cas13 assisted saliva‐based and smartphone integrated testing (CASSPIT), an RNA extraction‐free approach to detect SARS‐CoV‐2, which exhibited results comparable to those of the conventional RNA extraction method.^[^ ^65^ ^]^ In the SHERLOCK method, an extraction‐free protocol was achieved by treating saliva samples with phosphate‐buffered saline and 0.2% Triton X‐100, followed by heating at 95 °C for 5 min to release the target analytes.^[^ ^45^ ^]^ Subsequently, the heating unextracted diagnostic samples to obliterate nucleases (HUDSON) method was developed to rapidly detect multiple viruses, including Zika and dengue virus, by simply treating viral particles with TCEP/EDTA and heating.^[^ ^66^ ^]^ The streamlined highlighting of infections to navigate epidemics (SHINE) method developed by Arizti‐Sanz et al. uses HUDSON for sample pretreatment and a single‐step SHERLOCK assay to detect SARS‐CoV‐2 in saliva and nasopharyngeal swabs.^[^ ^67^ ^]^ Recently, a paper‐based extraction‐free method named miSHERLOCK (minimally instrumented SHERLOCK) has been shown to complete specimen filtration and enrichment, suggesting its potential for field‐applicable diagnosis.^[^ ^68^ ^]^ The low‐cost, high availability, and easy operation of extraction‐free methods are promising alternatives to conventional nucleic acid extraction tools suitable for use in portable and fully automated CRISPR‐based microfluidic devices.

## 3 Clustered Regularly Interspaced Short Palindromic Repeats/Clustered Regularly Interspaced Short Palindromic Repeats‐Associated Proteins Biosensing

**Figure 3:**
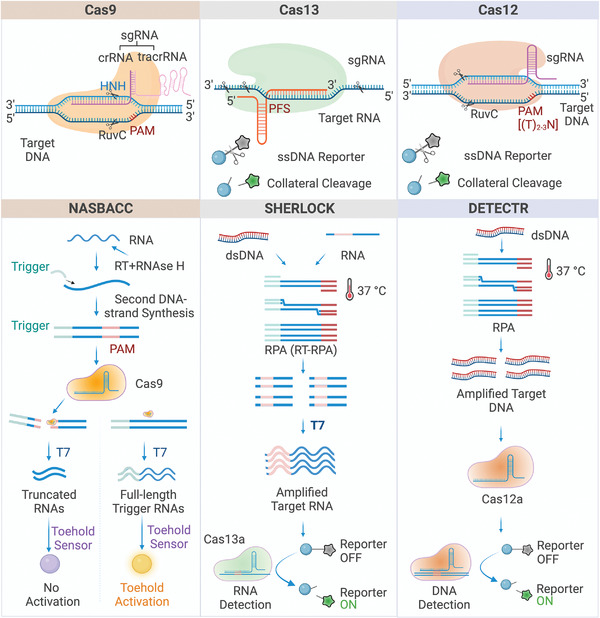
CRISPR‐based biosensing system. Three CRISPR‐based cleavage mechanisms (Cas9, Cas13, and Cas12) and three fundamental CRISPR‐based biosensing strategies (NASBACC, SHERLOCK, and DETECTR) are summarized.

CRISPR/Cas, an adaptive immune system in bacteria and archaea, recognizes foreign nucleotides that are further cleaved by Cas proteins with endonuclease activity.^[^ ^69^ ^]^ As a powerful gene manipulation tool, the CRISPR/Cas system has received widespread attention as a powerful tool for gene manipulation.^[^ ^70^, ^71^ ^]^ Moreover, due to its high programmability, specificity, and user‐friendliness, the CRISPR/Cas system has been utilized in the highly sensitive and selective molecular diagnosis with unprecedented success.^[^ ^64^, ^72^, ^73^ ^]^ In CRISPR/Cas systems, the pre‐CRISPR RNA (crRNA) is transcribed and processed into mature crRNA, which serves as the guide RNA for Cas effectors.^[^ ^74^ ^]^ These Cas effectors are composed of different types of single or complex proteins (Cas9, Cas12, and Cas13), which are critical for accurate cleavage in CRISPR‐based biosensing.^[^ ^75^ ^]^ As illustrated in **Figure** **3**, under the guidance of single‐guide RNA (sgRNA), Cas9 effectors can recognize and bind upstream of the 3’‐NGG protospacer adjacent motif (PAM) on target nucleic acids to activate the CRISPR/Cas9 system.^[^ ^76^, ^77^, ^78^ ^]^ The high hydrogen‐bonding affinity between PAM and Cas9 unwinds the target dsDNA, and HNH and RuvC‐like endonuclease cleave the complementary and noncomplementary chains, respectively.^[^ ^77^ ^]^ Comparatively, Cas13 and Cas12 effectors, which belong to type VI and type V proteins, respectively, nonspecifically cleave the target RNA (Cas13) or DNA (Cas12) because of their collateral effects.^[^ ^74^, ^79^ ^]^ Among these proteins, the Cas13 requires a specific sequence motif called the protospacer flanking site to empower flexible cleavage but displays satisfactory sensitivity. Cas12 requires only a sequence rich in T‐bases (5’‐TTTN‐3’) on the target, which significantly extends the application of the CRISPR/Cas12 system.^[^ ^74^, ^80^, ^81^, ^82^ ^]^ Typically, a fluorophore quencher (FQ)‐labeled reporter is introduced, which degrades and outputs a fluorescence signal because of the trans‐cleavage activity of Cas13/Cas12.^[^ ^74^, ^80^, ^81^, ^82^ ^]^ Moreover, the reporters can also be labeled by biotin‐FAM and generate colorimetric readouts.^[^ ^38^, ^62^, ^83^ ^]^

### 3.1 Cas9‐Based Biosensing Strategies

Cas9‐based biosensing strategies have attracted substantial attention for molecular diagnosis as pioneering Cas proteins. For example, in nucleic acid sequence‐based amplification‐CRISPR cleavage (NASBACC) (Figure 3), the CRISPR/Cas9 system collaborated with nucleic acid sequence‐based amplification (NASBA) to successfully detect Zika virus with femtomolar (fM) level sensitivity.^[^ ^64^ ^]^ In this study, the Cas9‐sgRNA complex could precisely cleave sequence‐specific DNA containing a 3’‐NGG PAM, which was employed to discriminate different viral lineages. Similarly, a CRISPR/Cas9 triggered isothermal exponential amplification reaction (CAS‐EXPAR) was designed to generate amplification primers by Cas9/sgRNA‐mediated cleavage activity, which selectively activated the amplification reaction by specific primer accumulation. The CAS‐EXPAR method detected Listeria monocytogenes bacterial DNA with a detection limit of 0.82 attomolar (aM) within 1 h.^[^ ^84^ ^]^ Considering that the Cas9‐sgRNA ribonucleoprotein complex exhibits low mismatch tolerance, particularly in PAM‐proximal mismatches, the CRISPR/Cas9‐triggered nicking endonuclease‐mediated strand displacement amplification method (CRISDA) was established to distinguish single‐nucleotide mismatches, which identified breast cancer‐associated homozygous and heterozygous genotypes with aM sensitivity.^[^ ^72^, ^85^, ^86^ ^]^

Furthermore, dead Cas9 (dCas9), an inactivated and mutated Cas9 effector, integrates with gRNA to form a ribonucleoprotein complex, which has also been utilized in CRISPR‐based infectious diseases diagnosis. For example, the dCas9‐based RCA‐CRISPR‐split‐HRP (RCH) method was established to detect target miRNAs with fM‐level sensitivity within 4 h and the clinical detection of circulating let‐7a, a reported biomarker of non‐small lung cancer.^[^ ^87^ ^]^ Another CRISPR/dCas9‐based colorimetric assay was designed to detect SARS‐CoV‐2 and the oseltamivir‐resistant pandemic influenza A (H1N1) virus with petamolar (pM)‐level sensitivity within 90 min.^[^ ^83^ ^]^ As previously confirmed, the ribonucleoprotein complex formed by the dCas9 protein and gRNA binds RNA with a biotin‐PAMmer with high programmability, which occurs in the streptavidin‐HPR/TMB visual streptavidin‐HPR/TMB reaction in microplate wells.^[^ ^88^ ^]^ Together with CRISPR/Cas collateral activity and the downstream sensing platform, detectable signals were further increased. As the first CRISPR‐based sensing technique was developed, various Cas9‐based approaches have been established for infectious diseases diagnosis.^[^ ^22^, ^64^, ^83^ ^]^

### 3.2 Cas13‐Based Biosensing Strategies

In addition to the cis‐cleavage activity analogous to the Cas9 system, the Cas12 and Cas13 systems also exhibit nonspecific trans‐cleavage activity toward the identified targets.^[^ ^11^ ^]^ The collateral activity of the Cas13‐crRNA complex was stimulated in the presence of target nucleic acids, which cleaved non‐targeted single‐stranded RNA modified by a fluorophore and quencher to generate a fluorescent signal for infectious diseases diagnosis.^[^ ^89^, ^90^ ^]^ In addition, collateral cleavage activity has been used as an internal signal for amplification, which outputs an amplified fluorescent signal for detection.^[^ ^34^, ^45^ ^]^ As shown in Figure 3, the first Cas13a‐based SHERLOCK system was developed as a brilliant tool that achieved an aM‐level detection and single‐base resolution.^[^ ^45^ ^]^ Using this method, the target nucleic acids were first amplified by recombinase polymerase amplification (RPA) for DNA or reverse transcription‐RPA (RT‐RPA) for RNA, followed by the catalytic substrate of T7 transcription to transform amplicons into RNA. The generated RNA was then recognized by a complex consisting of crRNA‐Cas13a, triggering its trans‐cleavage activity toward probes and output of a fluorescence signal that was utilized to sensitively detect Zika virus, dengue virus, resistance genes, and bacteria in clinical samples.^[^ ^45^ ^]^

Cas13‐based biosensing has been extensively studied for molecular detection, and ongoing efforts are being made to adapt it to the current public threat of infectious diseases. Recently, SHERLOCKv2, a modified version of SHERLOCK, has successfully transcended the SHERLOCK method into multiplex monitoring using visual lateral‐flow platforms, achieving aM sensitivity for Zika virus detection in human samples.^[^ ^34^ ^]^ Combined with the lateral‐flow readout of SHERLOCK assays, a HUDSON method was designed for on‐site detection that utilized heating and chemical reduction to complete the inactivation and lysis of raw samples in 30 min.^[^ ^91^ ^]^ The treated raw samples were directly mixed and reacted with CRISPR/Cas13a reaction reagents without requiring complete nucleic acid extraction and avoiding potential inhibitors. Using this method, the chemokine (C‐X‐C motif) ligand 9 (CXCL9) gene was detected with aM‐level sensitivity and single‐base resolution, facilitating POCT in resource‐limited regions. However, the methods mentioned above typically require multiple and separate operations to transfer amplicons, complicating diagnostic procedures and increasing the risk of contamination. Therefore, an ideal POCT method should be a fully integrated platform that combines sample pretreatment and CRISPR/Cas reactions into one‐pot or highly automated reactions. To achieve this goal, a sample‐in‐answer‐out and fully integrated method called SHINE was designed, in which RPA and Cas13a‐associated cleavage were completed in one step using the extraction‐free HUDSON protocol.^[^ ^14^ ^]^ This one‐step strategy produced fluorescent and lateral‐flow readouts in 50 min, and the limit of detection (LOD) values were 10 copies µL^−1^ and 100 copies µL^−1^, respectively. Considering that this Cas13‐based assay successfully detected SARS‐CoV‐2 RNA with high sensitivity and specificity, SHINE has the potential to be used for straightforward diagnosis outside hospitals.

**Figure 4:**
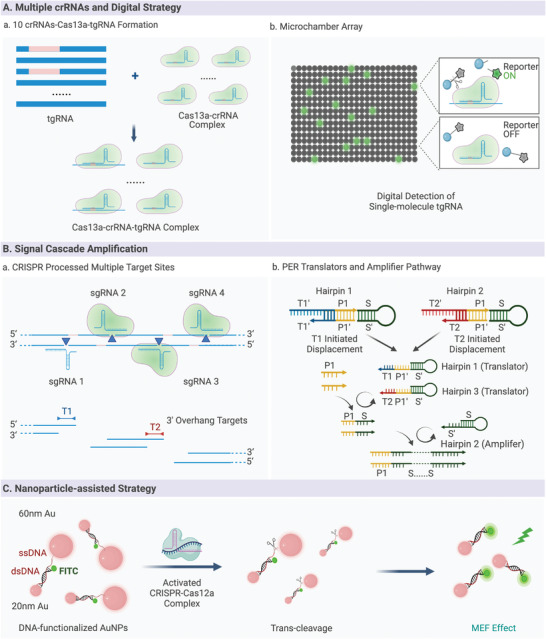
Amplification‐free CRISPR‐based biosensing. A) Multiple crRNAs and digital strategy. B) Signaling cascade amplification. C) Nanoparticle‐assisted strategy.

To achieve higher sensitivity, CRISPR/Cas biosensing systems generally need to be combined with pre‐amplification steps, such as PCR,^[^ ^92^ ^]^ RCA,^[^ ^93^ ^]^ RPA,^[^ ^64^ ^]^ and LAMP,^[^ ^62^ ^]^ but tend to prolong detection time and increase the risk of carryover contamination. Meanwhile, isothermal amplification may generate nonspecific amplicons, which further influence the specificity of diagnosis. Finally, the amplification process is nonlinear, limiting the quantitative detection of infectious diseases.^[^ ^45^, ^94^ ^]^ To overcome these limitations, various amplification‐free CRISPR‐based methods have been explored, such as multiple crRNA‐based determination,^[^ ^21^, ^95^ ^]^ signaling cascade amplification,^[^ ^96^, ^97^, ^98^ ^]^ and digital technology.^[^ ^20^, ^35^, ^61^, ^63^, ^92^, ^99^, ^100^ ^]^ It has been known that the CRISPR‐based amplification‐free digital RNA detection (SATORI) was designed with multiple crRNA strategies and CRISPR/Cas13a cleavage.^[^ ^95^ ^]^ As shown in **Figure** **4**A, this method creates 10 crRNAs of SARS‐CoV‐2 RNA, targeting 10 different regions in the N gene of SARS‐CoV‐2, and generates diverse LwaCas13a‐crRNA‐tgRNA triple complexes from a single target. When the reagents are divided into femtoliter (fL) sizes, each microchamber (*V* = 3 fL, *φ* = 2.5 µm, *h* = 0.6 µm) either contains or does not contain a single triple complex for a reaction. The total reaction system was completed within 5 min, reaching a detection limit of 5 fM using fluorescence microscopy. Furthermore, the signal cascade strategy was utilized in the CRISPR‐array‐mediated primer‐exchange‐reaction‐based biochemical circuit cascade, in which an autonomous signal converter was designed to link the upstream biochemical circuit with the downstream electrochemical readout (Figure 4B).^[^ ^98^ ^]^

### 3.3 Cas12‐Based Biosensing Strategies

Likewise, Cas12 has been widely applied in biosensing technologies with collateral activity, which does not require the in vitro transcription of amplified targets by T7 RNA polymerase necessary for Cas13‐based detection.^[^ ^74^, ^101^ ^]^ For example, CRISPR/Cas12a‐based one‐hour low‐cost multipurpose highly efficient system (HOLMES) was established to detect both the DNA virus (pseudorabies virus) and RNA virus (Japanese encephalitis virus). Combined with PCR amplification, HOLMES achieved aM sensitivity and single‐base variation within 1 h, and accurately distinguished single nucleotide polymorphism (SNP) genotypes or virus genotypes.^[^ ^102^ ^]^ However, PCR amplification in HOLMES requires time‐consuming thermal cycling. In the DETECTR method (Figure 3), RPA was combined with the LbCas12a effector to address this drawback and to realize rapid detection of highly pathogenic human papillomavirus (HPV) genotypes (HPV‐16 and HPV‐18).^[^ ^74^ ^]^ Compared to PCR, RPA maintains its stability and high efficiency at approximately 37 °C, which is more compatible with the CRISPR/Cas reaction.^[^ ^103^ ^]^ Similarly, Cas12a‐based visual detection (Cas12aVDet) was performed to conduct the entire test in a one‐pot system and further reduce the risk of aerosol contamination.^[^ ^104^ ^]^ All reagents except Cas12a were mixed and stirred together in a centrifuge tube, and Cas12a was dispersed on the inside wall. After the 15 min RPA reaction, the Cas 12a effector was transferred into the mixture through centrifugal force, which activated the downstream Cas12a cleavage activity and generated a fluorescence signal, achieving single‐molecule sensitivity within 30 min. This pioneering study has paved the way for rapid and portable molecular tests against SARS‐CoV‐2, thereby supporting the management of the COVID‐19 pandemic.

As shown in the studies mentioned above, the optimum cleavage activity of Cas12a occurs at ≈37 °C, and is reduced at temperatures exceeding 60 °C.^[^ ^105^, ^106^ ^]^ Compared to Cas12a, the optimal trans‐cleavage activity of Cas12b appears from 35 to 65 °C for ssDNA and from 45 to 55 °C for dsDNA, extending the application scenarios of CRISPR/Cas12‐based biosensing systems.^[^ ^107^ ^]^ Therefore, thermophilic Cas12b from Alicyclobacillus acidophilus (AapCas12b) is more compatible with LAMP or RT‐LAMP for target nucleic acid detection. Accordingly, HOLMESv2 and STOP (SHERLOCK testing in one pot) are upgraded technologies of this type.^[^ ^107^, ^108^ ^]^ HOLMESv2 can detect the Japanese encephalitis virus with a detection limit of 10 aM and distinguish DNA methylation and SNP discrimination in cell lines.^[^ ^107^ ^]^ Meanwhile, the STOP assay combining LAMP and Cas12b achieved the sensitive detection of SARS‐CoV‐2.^[^ ^108^ ^]^ Using this method, magnetic bead‐based cell lysis and nucleic acid purification shortened the extraction time to 15 min. This strategy reached an LOD of 2 copies µL^−1^ within 60 min when detecting SARS‐CoV‐2 using fluorescence or lateral‐flow readouts. Compared to the previous method based on Cas12a, the Cas12b enzyme is more thermally stable, which is compatible with a warm‐start to initiate the reaction in a homogeneous reaction system. This prevents premature target amplification at room temperature and enables the generation of a one‐step system for convenient target detection. Similarly, Cas12f can also target dsDNA and ssDNA but can better discriminate SNPs in ssDNA.^[^ ^11^, ^109^ ^]^

Research has focused on developing amplification‐free Cas12‐based biosensing systems and explore their application in infectious diseases diagnosis. Nanoparticle‐assisted strategies have been extensively developed toward better sensitivity, simplicity, and selectivity.^[^ ^110^, ^111^, ^112^, ^113^ ^]^ Given these characteristics, as shown in Figure 4C, ssDNA was degraded when the CRISPR/Cas12a complex was activated by target cell‐free DNA (cfDNA), resulting in DNA‐functionalized Au nanoparticles (AuNPs) producing a metal‐enhanced fluorescence signal. With a color change from purple to red‐purple, this nanoparticle‐assisted strategy enables highly sensitive detection of breast cancer gene‐1 within 30 min.^[^ ^110^ ^]^

## 4 Clustered Regularly Interspaced Short Palindromic Repeats‐Based Microfluidic Diagnosis

Based on the fusion of multidisciplinary approaches,^[^ ^114^, ^115^ ^]^ microfluidic devices have been extensively explored as tools for the separation or manipulation of substrates,^[^ ^17^ ^]^ in which researchers can manipulate small amounts of liquid (10^−9^ to 10^−18^ L) within elaborate channels of micrometer (10–100 µm) sizes.^[^ ^114^ ^]^ This particular structure and dynamic design have revealed the following advantages: 1) Automated detection with faster turnaround times, 2) cost‐effectiveness with smaller samples and reagent volume requirements (µL–pL), 3) high‐throughput detection with simultaneous analysis of multiple targets, and 4) field‐deployable diagnosis with simplified and integrated operations.^[^ ^114^, ^116^, ^117^ ^]^ Because of these advantages, microfluidic devices have been developed for molecular diagnosis.^[^ ^17^ ^]^ The combination of microfluidic technology and highly integrated CRISPR‐based biosensing shows excellent potential for multiplexed and field‐deployable possibilities. Here, we briefly summarize the latest achievements in microfluidic CRISPR/Cas systems for infectious diseases diagnosis, which have significantly contributed to the development of CRISPR‐based biosensing strategies (**Table** **1**).

**Table 1:** Summary of CRISPR‐based microfluidic device

Name^a),b)^	Cas	Target	Organism	Material	Amplification	Readout	Assay Time [min]	LOD	Quantitative	Multiplex	Clinical Sample	Reference
Polymer‐based CRISPR microfluidic devices												
dCas9 assay	dCas9	RNA	SARS‐CoV‐2, pH1N1	PDMS	‐^b^	Colorimetric	90	pM	N	Y	NP swab, sputum	[83]
Automated chip	LwCas13a	RNA	EBOV, Marburg	PDMS	‐	Fluorescence	5	5.45 × 10^4^ copies µL^−1^	Y	YP	Blood	[28]
Electrochemical biosensor	Cas13a	RNA	miRNA	Foil, polyimide	‐	Electrochemical	240	10 pM	Y	N	Serum	[96]
IMPACT	LbCas12a	DNA	ASFV	PDMS	‐	Fluorescence	120	1 nM	N	Y	/	[118]
All‐solution phase assay	LbCas12a	DNA	ASFV	PDMS	‐	Fluorescence	120	1 pM	Y	N	/	[119]
MXene‐PEDOT	LbCas12a	DNA	HPV	PDMS	‐	Fluorescence	30	15.22 pM	N	N	/	[120]
Raman‐sensitive assay	Cas12a	DNA	HBV, HPV (16.18)	PDMS	‐	Surface‐enhanced Raman scatting	20	1 aM, 100 aM	Y	Y	/	[111]
CASMEAN	AsCas12a, LbCas12a	DNA	*P. aeruginosa*	PMMA	RAA	Fluorescence	90	1 aM	Y	N	/	[121]
Mobile phone‐based assay	LbuCas13a	RNA	SARS‐CoV‐2	PDMS	‐	Fluorescence	30	100 copies µL^−1^	Y	N	NP swab	[21]
CASCADE	LbaCas12a	RNA	SARS‐CoV‐2	PMMA	‐	Fluorescence	71	50 copies µL^−1^	Y	N	NP swab	[122]
Paper‐based CRISPR microfluidic devices												
Toehold switches	Cas9	RNA	Zika virus	Paper	RPA	Colorimetric, Fluorescence	60–180	3 fM	N	Y	Plasma	[64]
SHERLOCK	LwaCas13a	DNA/RNA	SARS‐CoV‐2	Paper	RPA/RT‐RPA	Fluorescence	120–300	3 copies µL^−1^	N	Y	NP/throat swabs	[45]
SHERLOCK v2	LwaCas13, PsmCas12, Cas13b	DNA/RNA	Zika, Dengue, *E. coli*, *K. pneumoniae*	Paper	RPA/RT‐RPA	Fluorescence, colorimetric	30–180	2 aM	Y	Y	Urine, saliva	[34]
µPad CRISPR	LbCas12a	RNA	EBOV	Paper	RT‐RPA	Fluorescence, digital signal	60–240	11 aM	Y	Y	PBMCs	[123]
HUDSON‐SHERLOCK	LwaCas13a	DNA/RNA	EBOV, LASV‐II, LASV‐IV	Paper	RPA/RT‐RPA	Fluorescence, colorimetric	60	10 copies µL^−1^, 10 copies µL^−1^, 100 copies µL^−1^	Y	N	Blood, urine, saliva	[13]
SHIINE	LwaCas13a	RNA	SARS‐CoV‐2	Paper	RT‐RPA	LFR	50	100 copies µL^−1^	N	N	NP swab	[14]
DETECTR	LbCas12a	RNA	SARS‐CoV‐2	Paper	RT‐LAMP	Fluorescence	30–40	10 copies µL^−1^	N	N	NP/OP swab	[12]
STOPCovid.v2	AapCas12b	RNA	SARS‐CoV‐2	Paper	LAMP	Fluorescence, LFR	60	2 copies µL^−1^	N	N	NP swab	[108]
CASLFA	Cas9	DNA	LM, ASFV	Paper	RPA	LFR	≈38	150 copies	N	N	Swine serum	[38]
VaNGuard	Various Cas12a	RNA	SARS‐CoV‐2	Paper	RT‐LAMP	Fluorescence, LFR	25–30	8 copies µL^−1^	N	N	NP swab, saliva	[124]
Autonomous assay	Cas12a	RNA	SARS‐CoV‐2	Paper	RT‐RPA	Fluorescence	60	100 copies	N	N	NP swab	[40]
TL‐LFA	Cas9	RNA	SARS‐CoV‐2	Paper	RT‐RPA	Fluorescence	58	100 copies	N	N	NP swab	[22]
µReaCH‐PAD	LbaCas12a	RNA	Candida, Aspergillus	Paper	RT‐RAA	Colorimetric	90	4.90, 4.13 CFU mL^−1^	Y	Y	Ascites, wound discharge, urine, blood, and sputum	[18]
CIA	LbaCas12a, AaCas12b	DNA	P. aeruginosa, *E. coli*	Paper	LAMP	Fluorescence	50	1 copy, 1 CFU mL^−1^	N	N	Recombinant Plasmid	[62]
CRA‐LFB	LbaCas12a	DNA	S. aureus	Paper	RAA	Fluorescence	70	540 CFU mL^−1^	N	N	Natural meat, vegetable	[41]
Self‐contained Chip	LbCas12a	RNA	SARS‐CoV‐2	Paper	RT‐RPA	LFR	60	100 copies	N	N	NP swab	[125]
Electronic CRISPR microfluidic devices												
E‐CRISPR	LbCas12a	DNA	parvovirusB19, HPV‐16	PET	‐	Electrochemical	30	50 pM	Y	N	Spike sample	[126]
E‐DNA	Cas9, Cas12a	DNA	parvovirus B19	PET	‐	Electrochemical	30	100 fM	Y	N	/	[127]
PER‐based cascade	Cas9	DNA	SARS‐CoV‐2	PET	‐	Electrochemical	90	5–200 nM	Y	N	Human cell lysates	[98]
ITP‐CRISPR	LbCas12a	DNA/RNA	SARS‐CoV‐2	Glass	LAMP/RT‐LAMP	Electrochemical	35	10 copies µL^−1^	Y	N	NP swab	[57]
“On–off” sensor	Cas12a	DNA	HPV‐16, HIV	GE	‐	Electrochemical	120	0.32 pM	Y	Y	Serum, swab	[113]
Digital CRISPR microfluidic devices												
Ultralocalized assay	Cas13a	RNA	SARS‐CoV‐2	PDMS	‐	Fluorescence	70	10 aM	Y	N	NP swab	[20]
CARMEN v.1	LbuCas13	RNA	169 viruses; subtyping of influenza A strains	PDMS	PCR/RPA	Fluorescence	200	540 copies µL^−1^	Y	Y	Plasma, serum, throat, nasal swab	[92]
Dual‐crRNA assay	LbCas12a	DNA	ASFV, EBV, HBV	PDMS	‐	Fluorescence	60	1.75 copies µL^−1^	Y	N	Serum	[99]
deCOViD	LbCas12a	RNA	SARS‐CoV‐2	silicon	RT‐RPA	Fluorescence	15–30	1 copy µL^−1^	Y	N	NP swab	[46]
One‐step assay	LbaCas12a	Cell‐free DNA	HBV, HPV (16,18)	PDMS	‐	Fluorescence	30	5 fM	Y	Y	Serum	[128]
SATORI	LwaCas13a	RNA	SARS‐CoV‐2	Polymer, glass	‐	Fluorescence	5	5 fM	Y	Y	NP, anterior nasal, throat swab	[95]
mCARMEN	LbuCas13	RNA	Respiratory viruses	IFC	PCR/RPA	Fluorescence	100	10 copies µL^−1^	Y	Y	Plasma, serum, throat, nasal swab	[100]
DropCRISPR	Cas12a	DNA	St	PDMS	LAMP	Fluorescence	60	100 CFU mL^−1^	Y	N	Raw milk samples	[129]
Wearable CRISPR microfluidic devices												
wFDCF Assay	LbaCas12a	DNA	MRSA	Textiles, polymer, paper	RPA	Fluorescence	56–78	aM	N	Y	/	[15]
Face‐mask‐integrated sensor	LbaCas12a	RNA	SARS‐CoV‐2	Paper	RT‐RPA	Fluorescence	90	17 aM	N	N	Exhaled aerosols	[15]
Wearable microneedles	dCas9	DNA	EBV	PDMS	‐	Electrochemical	30	1.1 fM	Y	N	/	[130]

^a)^ “Y” represents Yes, “N” represents No, “‐” means this method does not need signal amplification strategies such as LAMP, PCR, RCA, or RPA

^b)^ dCas9, dead Cas9; LFR, Lateral Flow Readout; EBOV, Ebola virus; LASV, Lassa virus; ASFV, African swine fever virus; IF, Invasive fungi; P. aeruginosa, Pseudomonas aeruginosa; S. aureus, Staphylococcus aureus; EBV, Epstein‐Barr virus; HBV, Hepatitis B virus; MASA, Methicillin‐resistant Staphylococcus aureus; LM, Listeria monocytogenes; St, Salmonella typhimurium; RT‐RPA, reverse transcription recombinase polymerase amplification; RT‐LAMP, reverse transcription loop‐mediated isothermal amplification; RT‐RAA, reverse transcription recombinase‐ aided amplification; LbCas12a, Cas12a from Lachnospiraceae bacterium ND2006; AsCas12a, the Acidaminococcus sp. Cas12a; LwCas13a, Cas13a from Leptotrichia wadei; PBMCs, human primary peripheral blood mononuclear cells; PET, polyethylene terephthalate; GE, serum and swab samples; PER‐based, primer exchange reaction‐based; NP, nasopharyngeal; OP, oropharyngeal; IFC, Fluidigm integrated fluidic circuit.

### 4.1 Polymer‐Based Microfluidic Devices

Microfluidic device fabrication is a critical step in integrated automated CRISPR/Cas biosensing. Various materials, including polymers, silicon, metals, glass, hydrogels, and paper, have been used to fabricate microfluidic devices.^[^ ^131^ ^]^ Among these, polymers have attracted increasing attention owing to their low price, well‐developed fabrication protocol, and high biocompatibility.^[^ ^17^ ^]^ In particular, polydimethylsiloxane (PDMS) is widely used because of its unique advantages, including high biocompatibility of reactions,^[^ ^132^ ^]^ transparency for observations,^[^ ^133^ ^]^ gas permeability for diffusion,^[^ ^134^ ^]^ and soft lithography method for manufacturing, which is relatively easy.^[^ ^17^ ^]^ Polymethyl methacrylate (PMMA), similarly, owns the advantages of an affordable cost, good optical and mechanical properties.^[^ ^135^ ^]^

**Figure 5:**
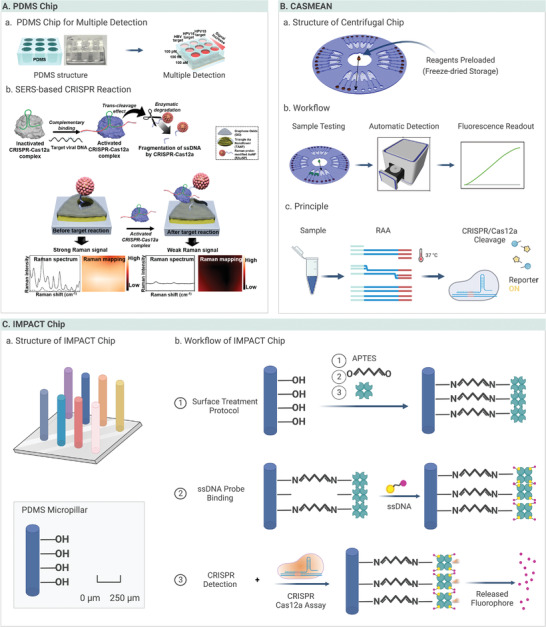
Polymer‐based microfluidic devices. A) The illustration of CRISPR‐based surface‐enhanced Raman spectroscopy‐active nanoarray. Reproduced with permission.^[^ ^111^ ^]^ Copyright 2021, American Chemical Society. B) The illustration of CASMEAN. Reproduced with permission.^[^ ^121^ ^]^ Copyright 2020, American Chemical Society. C) The description of the IMPACT chip. The chip comprises a PDMS micropillar with surface treatment and ssDNA probe binding to achieve CRISPR detection.

Recently, innovations in polymer‐based microfluidics combined with CRISPR‐based biosensing have attracted considerable attention for the detection of infectious diseases.^[^ ^118^ ^]^ Namely, a CRISPR/Cas13a‐based amplification‐free method integrated mobile phone microscopy on a PDMS chip to detect SARS‐CoV‐2 RNA as low as 100 copies µL^−1^ in 30 min.^[^ ^21^ ^]^ Combined with surface‐enhanced Raman sensitive spectroscopy (SERS) technology, CRISPR‐based microfluidic biosensing achieved multiplexed and amplification‐free detection (**Figure** **5**A).^[^ ^111^ ^]^ A SERS‐active graphene oxide (GO)/triangular Au nanoflower (GO‐TANF) with a Raman probe‐modified Au nanoparticle (RAuNP) was designed to increase the SERS signal. When the CRISPR/Cas12a complex was activated in the presence of target analytes, the ssDNA was trans‐cleaved, resulting in the separation of the pro‐immobilized RAuNP, which decreased the SERS signal. Together with these innovations, HPV‐16, HPV‐18, and HBV were simultaneously detected, with LODs ranging from 1 aM to 100 pM. Compared to other systems, this polymer‐based microfluidic system improved multi‐target detection sensitivity with an extremely low detection limit. This system is also one of the most ingenious recent examples of ultrasensitive amplification‐free detection platforms, which integrates several techniques and provides a simple and ready‐to‐use detection approach because it does not require any pre‐steps (nucleic acid amplification) or post‐steps such as labeling and washing after the target reaction.

Significant progress has been achieved in the development of stand‐alone microfluidic systems for both automatic processing and signal output. Typically, pneumatically controlled devices were established for 24 assays in parallel using automated polymer‐based CRISPR microfluidics.^[^ ^28^ ^]^ Following this method, a parabolic mirror‐based fluorometer was designed in a Cas13a‐based microfluidic system to detect DNA and RNA pathogens with fM‐level sensitivity without amplification, reaching an LOD of 20 PFU mL^−1^ within 5 min when detecting Ebola RNA. Chen et al. designed a Cas12a‐assisted straightforward microfluidic device for the analysis of nucleic acids (CASMEAN) (Figure 5B), in which the reagents were prestored in centrifugal microfluidic devices and freeze‐dried for automatic RAA‐Cas12a detection.^[^ ^121^ ^]^ Using this strategy, the automated chip was controlled by sequential liquid operations from inward to outward, enabling the rapid and convenient detection of nucleic acids within 1.5 h.

As shown in Figure 5C, an integrated micropillar polydimethylsiloxane accurate CRISPR detection (IMPACT) strategy was established in which the enclosed PDMS microchannel was coated beforehand with streptavidin as a probe. With collateral cleavage of the Cas12a complex, micropillars release large amounts of fluorescence reporters proportional to the target DNA ranging from 0.1 to 1 nM.^[^ ^118^ ^]^ Sliva et al. reported a cellphone‐based amplification‐free system with CRISPR/Cas‐dependent enzymeatic (CASCADE) to detect SARS‐CoV‐2 RNA. In this assay, PMMA microfluidics were used for CRISPR‐based detection, where catalase‐generated gas bubble fluorescence signals were imaged using a cellphone camera.^[^ ^122^ ^]^ Without pre‐amplification, this strategy could detect target analytes at concentrations as low as 50 copies µL^−1^ in 71 min. Although these assays require a larger sample size to validate their applicability, they have demonstrated great promise for bringing polymer‐based microfluidic devices into infectious diseases detection. The chip comprised a PDMS micropillar with surface treatment and ssDNA probe binding to achieve CRISPR detection.

### 4.2 Paper‐Based Microfluidic Devices

**Figure 6:**
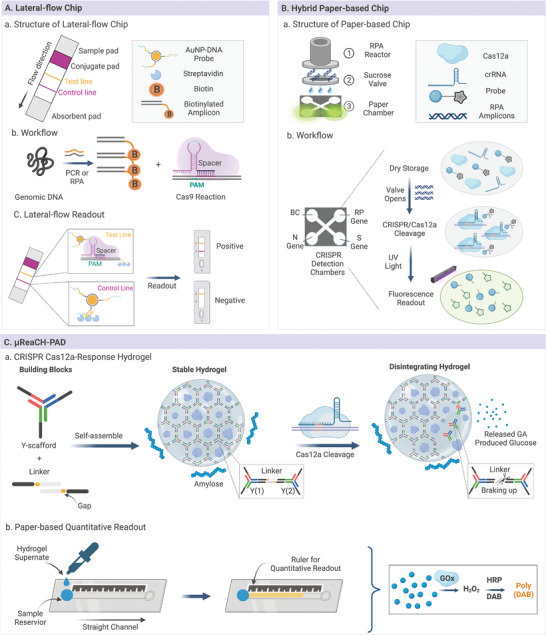
Paper‐based microfluidic devices. A) Workflow of advanced CASLFA method and construction of the lateral‐flow chip. B) Device constitution and workflow of hybrid paper‐based CRISPR chambers for multiplex gene diagnosis. C) Device constitution and workflow of µReaCH‐PAD.

As an alternative to conventional microfluidic substrates (such as PDMS, glass, plastics, and silicon), paper‐based microfluidics can comprise hydrophilic and hydrophobic microstructures through modifications using techniques such as photolithography, wax printing, and chemical vapor‐phase deposition.^[^ ^136^ ^]^ Paper‐based microfluidics exhibit the following unparalleled advantages: 1) Ease of manufacture and low cost, which is suitable for limited‐resource settings; 2) self‐driven by capillary action without external force; 3) portability and flexibility for field‐deployable applications; and 4) application of biodegradable and environmentally friendly microfluidics that can be incinerated after use.^[^ ^137^ ^]^ As a unique platform, paper‐based CRISPR/Cas microfluidic detection has been developed for infectious diseases diagnosis.^[^ ^22^, ^38^, ^40^, ^62^, ^138^, ^139^, ^140^, ^141^ ^]^ We briefly divided paper‐based CRISPR microfluidics into lateral flow assays (LFAs) and other paper‐based assays based on their fundamental properties. In addition to immunoassays, LFA strategies for the diagnosis of infectious diseases have been widely promoted.^[^ ^142^ ^]^ Combined with CRISPR‐based cleavage activity, LFA testing may exhibit extremely high specificity compared to traditional test strips.^[^ ^143^ ^]^ Specifically, in a novel diagnostic tool called CRISPR/Cas9‐mediated lateral flow assay (CASLFA), a universally used sgRNA was designed to recognize AuNP‐DNA probe‐Cas9/sgRNA‐biotinylated amplicons (**Figure** **6**A).^[^ ^38^ ^]^ This test strip could detect SARS‐CoV‐2 and African swine fever virus with a detection limit of 100 copies within 1 h. Subsequently, a CRISPR/Cas9‐mediated LFA was developed to increase the specificity and accuracy of CASLFA, which achieved simultaneous dual‐gene (envelope and open reading frame 1ab genes) diagnosis of SARS‐CoV‐2 infection.^[^ ^22^ ^]^ Additionally, the HUDSON‐SHERLOCK assay was designed to facilitate the Ebola and Lassa fever tests in Sierra Leone and Nigeria at a low cost (one dollar per sample), which significantly contributed to the field‐deployable and affordable applications of LFAs.^[^ ^13^, ^45^ ^]^ Li et al. introduced a self‐contained CRISPR microfluidic chip with a warmer‐powered heater case and a lateral flow dipstick to alleviate substrate contamination and potential false‐positive results in LFAs.^[^ ^34^ ^]^ This completely‐closed platform integrated the RT‐RPA reaction, CRISPR diagnosis with prestored reagents, and lateral flow readout, detecting as low as 100 copies of SARS‐CoV‐2 RNA per test.^[^ ^125^ ^]^

In addition to LFAs, other paper‐based CRISPR microfluidics have recently been developed. These paper‐based analytical devices have two different 3D structures. One is a 2D device patterned with hydrophobic borders as channels, and the other is a 3D device fabricated by paper folding, 3D printing, and slip techniques.^[^ ^144^ ^]^ For example, our group developed an autonomous CRISPR/Cas microfluidic detection method composed of a 3D‐printed pre‐amplification chamber, a paper‐based sucrose valve, and paper‐based CRISPR/Cas detection chambers printed and separated by black wax for multiplex diagnosis (Figure 6B). The optimized sucrose concentration ensured autonomous initiation of the downstream CRISPR reaction after the RPA reaction was completed, which could detect 100 copies of SARS‐CoV‐2 RNA per test within 1 h.^[^ ^40^ ^]^ However, quantitative detection remains a major challenge in paper‐based microfluidics. To solve this problem, Huang et al. designed µReaCH‐PAD (microfluidic ruler‐readout and CRISPR Cas12a‐responded hydrogel‐integrated paper‐based analytical devices) to detect invasive fungi (Figure 6C) quantitatively.^[^ ^18^ ^]^ This wax‐patterned paper chip consists of a circular reservoir to load samples, a straight channel for quantitative reactions, and several equal scales for visible signal readout. When activated CRISPR/Cas12a disintegrates the hydrogel structure and releases glucose onto the paper chip, the preloaded glucose oxidase (GOx) oxidizes glucose into H_2_O_2_, converting DAB to brown poly (DAB) for visible readout. The quantitative detection of invasive fungi from 10 to 10^4^ CFU mL^−1^ was performed using this cascade of enzymatic reactions with reasonable specificity and accuracy. In summary, these paper‐based CRISPR microfluidics have shown great promise for meeting the critical needs of POCT challenges currently encountered in resource‐limited settings with increasing sensitivity and stability.

### 4.3 Electronic Microfluidic Devices

Electronic microfluidic biosensors are efficient biosensing strategies in which the electronic transducer transforms biochemical signals and depends on the enzymatic catalysis of immobilized biomolecules to generate electrons that influence the electrical properties and output electronic signals.^[^ ^145^, ^146^ ^]^ Electronic microfluidic biosensors have been widely used for molecular diagnosis owing to their rapid analysis efficiency, excellent selectivity, cost‐effectiveness, user‐friendliness, and real‐time monitoring ability.^[^ ^147^, ^148^ ^]^ The specific and programmable RNA‐guided cleavage activity of the CRISPR/Cas system makes it a potential biorecognition tool that can be utilized for target recognition and cleavage of electrochemical tags on the surface of the sensor.^[^ ^74^, ^127^ ^]^ As a result, electronic CRISPR microfluidic biosensors will increase the possibility of next‐generation, field‐deployable diagnostic devices.^[^ ^149^ ^]^

**Figure 7:**
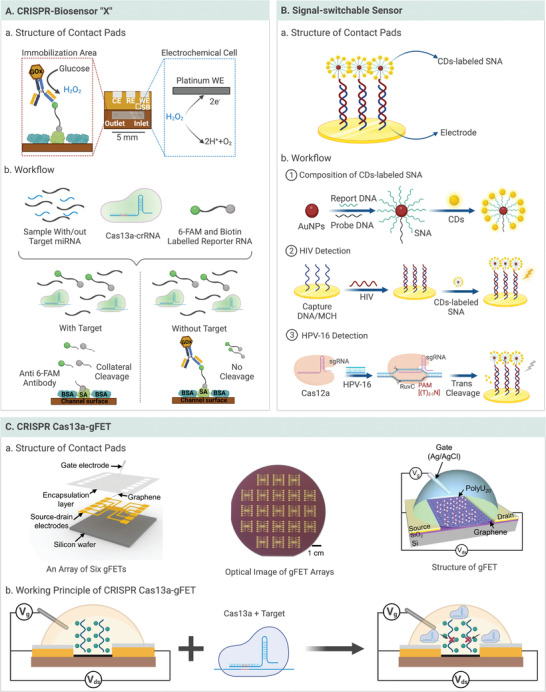
Electronic CRISPR microfluidic devices. A) Illustration of CRISPR‐biosensor “X” and the workflow on this electrode. B) Illustration of “on–off” signal‐switchable sensor and the workflow on this chamber. C) Illustration of CRISPR Cas13a‐gFET. Reproduced with permission.^[^ ^150^ ^]^ Copyright 2022, Wiley‐VCH GmbH.

Recently, a series of electronic CRISPR‐based microfluidic devices has been developed for infectious diseases diagnosis.^[^ ^96^, ^97^, ^98^, ^112^, ^113^, ^126^, ^127^, ^148^ ^]^ For example, a CRISPR‐array‐mediated primer‐exchange‐reaction (PER)‐based biochemical circuit cascade was designed based on the effect of signaling cascade amplification to detect the SARS‐CoV‐2 genome with the nanomolar (nM)‐level sensitivity in 90 min.^[^ ^98^ ^]^ This surface‐modified electronic sensor in a microfluidic device was shown to be stable during long‐term dry storage.^[^ ^126^ ^]^ Another CRISPR/Cas12a‐based electrochemical biosensor (E‐CRISPR) was designed to diagnose HPV‐16 and parvovirus B19 with picomolar (pM) sensitivity.^[^ ^126^ ^]^ Furthermore, a methylene blue (MB)‐based electrochemical DNA (E‐DNA) sensor was developed (**Figure** **7**A).^[^ ^127^ ^]^ In this method, hairpin ssDNA was immobilized with MB on an electrochemical effector to reduce the distance between the electrodes and ssDNA. In the presence of the target analytes, the trans‐cleavage activity of Cas12a‐crRNA was released from the electrochemical effector and generated a low electrochemical current, which achieved fM sensitivity without enzymatic amplification. Moreover, potential mismatches in the target sequence can be distinguished using this method, which can determine a single mutation compared with the classic DNA electrochemical method.

Additionally, electronic CRISPR/Cas microfluidic devices have exhibited the ability to perform multiplex molecular diagnosis of infectious diseases. As illustrated in Figure 7B, an “on–off” signal‐switchable electrochemiluminescence (ECL) biosensor was reported to detect HIV and HPV‐16 simultaneously.^[^ ^113^ ^]^ In this study, ECL luminophores of carbon dot (CD)‐labeled spherical nucleic acid (SNA) were captured on the biosensor with HIV DNA, resulting in an “on” signal. In the presence of HPV‐16 dsDNA, the Cas12a effector trans‐cleaved the specific ssDNA sequence from CD‐labeled SNA, hindering the aforementioned capture and resulting in an “off” signal. Using this strategy, HPV‐16 and HIV were successfully and quantitatively detected with 30 fM and 0.32 pM sensitivity within 2 h, respectively.

Furthermore, the cooperativity of Cas13a trans‐cleavage activity and ultrasensitive graphene field‐effect transistors (gFET) was utilized to detect SARS‐CoV‐2 and respiratory syncytial virus genome with 1 aM sensitivity in 30 min (Figure 7C).^[^ ^150^, ^151^ ^]^ As a three‐terminal electronic device, a field‐effect transistor (FET) enables the gate electrode to produce an external electric field, which can control the source‐drain current across the semiconductor channel.^[^ ^152^ ^]^ Upon multi‐turnover trans‐cleavage of Cas13a introduced by the target sequence, the charge neutrality point voltages (*V*_CNP_) shifted positively, resulting in the cleavage of a negatively charged RNA reporter (PolyU_20_) and decreased electron transference from the RNA to the graphene channel. Quantitative detection of the target RNA was achieved by measuring the magnitude of the *V*_CNP_ shift associated with the target concentrations. In addition, the developed CRISPR‐Chip could capture and detect target DNA by relying on catalytically RNA‐guided deactivated Cas9 immobilized on an analogous gFET transistor. In this method, anchored dCas9 enzymes can sensitively scan, bind, and enrich the target sequence, achieving 1.7 fM within 15 min for clinical samples from individuals with Duchenne muscular dystrophy.^[^ ^153^ ^]^ In conclusion, electronic microfluidic devices take advantage of accurate CRISPR/Cas‐based cleavage and ingenious electrochemical signal outputs to achieve ultrasensitive detection. Thus, electronic CRISPR/Cas‐based microfluidic detection is a promising modality for meeting the requirements of POCT for infectious diseases.

### 4.4 Digital Microfluidic Devices

Digital bioassays are highly compatible and highly efficient detection strategies in which reaction reagents are partitioned into numerous microreactors, and each compartment can be loaded separately with or without target analytes.^[^ ^154^, ^155^ ^]^ In digital bioassays, two strategies have been widely employed for precise boundary solutions: droplet microfluidics and microchamber arrays. In droplet microfluidics, droplets are formed continuously by the extrusion and shearing of two different insoluble media in a microchannel, controlled by regulating the flow rate ratios and channel dimensions.^[^ ^114^, ^156^ ^]^ While in microchamber arrays, the reagents are transported to each microchamber through a single pipeline spanning.^[^ ^157^ ^]^ Accurate quantification of digital microfluidics ensured that each target nucleic acid had the same probability of participating in the reaction system. Additionally, when integrated with CRISPR biosensing, this universal, quantifiable, and straightforward microfluidic device has captured increasing attention for infectious diseases diagnosis.^[^ ^20^, ^36^, ^63^, ^100^ ^]^

**Figure 8:**
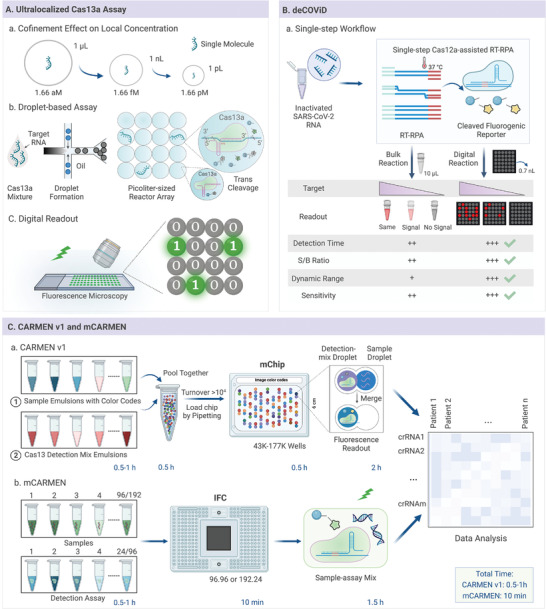
Digital microfluidic devices. A) Schematic of the ultralocalized Cas13a assay on microfluidic droplet chip. B) Illustration of deCOViD. C) Schematic and comparison of CARMEN v.1 and mCARMEN workflow.

Compared to traditional molecular diagnostic methods, digital CRISPR‐based microfluidic detection exhibits a more automatic and highly integrated structure with high sensitivity and fewer reagents. Droplet microfluidics is appropriate for analyzing single molecules and cells.^[^ ^99^, ^158^ ^]^ Tian et al. combined the droplet microfluidics and CRISPR‐based cleavage activity to achieve the single‐molecule quantification and diagnosis of 16S rRNA, microRNA, and SARS‐CoV‐2 (**Figure** **8**A).^[^ ^20^ ^]^ Additionally, the RApid Digital Crispr Approach (RADICA) and digital‐enhanced CRISPR/Cas‐assisted one‐pot virus detection (deCOViD) (Figure 8B) could be completed without requiring tedious and costly PCR thermocyclers.^[^ ^39^, ^46^ ^]^ Increasing research attention has been focused on integrated RT‐RPA/RPA amplification and CRISPR‐based biosensing into digital chips for the single‐molecule quantification of pathogens. However, this one‐pot deCOViD method may overestimate the initial targets, as the gentle reaction temperature used for RPA/RT‐RPA may cause target pre‐amplification.^[^ ^159^ ^]^ To address this challenge, Ding et al. introduced a digital warm‐start CRISPR assay to solve this problem, which applied Bst DNA polymerase‐based isothermal amplification (DAMP/RT‐DAMP) and Cas12a effectors in a one‐pot format.^[^ ^61^ ^]^ This method can directly detect nucleic acids from raw samples using a simple heating process, reaching an LOD of 5 copies µL^−1^ SARS‐CoV‐2 RNA on‐chip within 90 min.

Multiplexed infectious diseases diagnosis in digital CRISPR‐based microfluidics is another research hotspot that is mainly achieved by labeling and encoding. Signal molecules can distinguish different analytes in the labeling strategy, such as quantum dots or organic dyes.^[^ ^99^ ^]^ In comparison, the encoding scheme facilitates massive multiplex detection, avoiding spectral overlap and potential errors. For example, self‐organizing and miniaturized digital microfluidic technologies have been utilized to scale up the capabilities of the encoding strategy in CARMEN v.1 (Figure 8C).^[^ ^160^, ^161^, ^162^ ^]^ After color coding and emulsification, the amplicons were self‐organized and paired with CRISPR/Cas13 reagents in droplets of the microwell array. Although CARMEN v.1 mainly relies on customized imaging chips, readout hardware, and a low‐throughput workflow of 8–10 h, this powerful tool could concurrently detect 169 human‐related viruses in eight samples, identify different influenza A subtypes, and distinguish HIV drug‐resistant variants.^[^ ^92^, ^163^ ^]^

To fulfill the urgent need for clinically relevant surveillance related to multiple detections, microfluidic combinatorial arrayed reactions for multiplexed evaluation of nucleic acids (mCARMEN) have been developed, which use commercial microfluidics to detect 21 viruses, including SARS‐CoV‐2 and influenza strains, with excellent sensitivity (Figure 8C).^[^ ^100^ ^]^ Additionally, an mCARMEN variant identification panel (VIP) was designed to identify six SARS‐CoV‐2 variants, such as Delta and Omicron, and achieved approximately 100% consistency compared with time‐consuming and expensive NGS when VIP was used to analyze 2088 patient specimens.^[^ ^164^, ^165^ ^]^ Therefore, mCARMEN, a facile integrated platform, has the potential to monitor multiple respiratory viruses in one day and quantify their genomic copies, which is vital for early diagnosis of infectious diseases and effective decision making. Additionally, multiplexed intermixed CRISPR droplets (MIC‐Drop), a digital microfluidic technology, have been established for large‐scale in vivo CRISPR screens.^[^ ^166^ ^]^ The strong versatility of this platform enables in vivo vertebrate CRISPR screens with full potential to extend to broader research fields. Moreover, considering the advantages of 3D printing, including ease of operation, allowance for testing‐based optimization, and unique device architecture, digital microfluidic devices combined with 3D printing technology can be used for portable and ultrasensitive detection.^[^ ^128^, ^129^, ^167^ ^]^

### 4.5 Wearable Microfluidic Devices

Currently, clinical samples are collected from blood, serum, or nasopharyngeal swabs, and preserved in sterile containers for infectious diseases diagnosis.^[^ ^168^, ^169^, ^170^ ^]^ Despite the increased sensitivity and rapid diagnostic techniques, these methods still require the destruction of the skin or mucous membranes and cause trauma, associated with an unpleasant diagnostic experience and potential risk of infection.^[^ ^171^ ^]^ Therefore, noninvasive diagnostic methods are preferred as alternatives for patients in intensive care units who require long‐term detection or susceptible populations, such as patients with autoimmune diseases.^[^ ^172^ ^]^ As a transcendent non‐invasive detection strategy, wearable biosensors are particularly attractive and can be implanted in clothing, skin, or the body.^[^ ^173^ ^]^ Moreover, regarding the strength of wireless data transmission and micropower modules, sensing data can be collected using computers or cell phones, which are suitable for follow‐up health analyses.^[^ ^174^ ^]^

**Figure 9:**
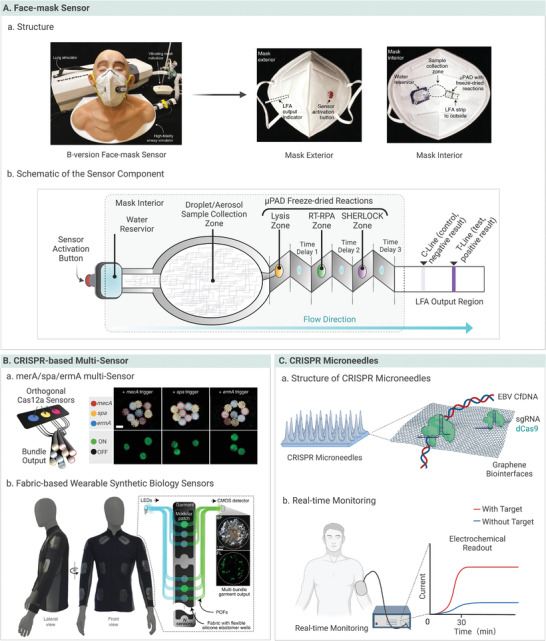
Wearable microfluidic devices. A) The facemask‐integrated SARS‐CoV‐2 wearable device and the schematic of the SARS‐CoV‐2 sensor components. Reproduced with permission.^[^ ^15^ ^]^ Copyright 2021, Springer Nature Limited. B) CRISPR‐based multi‐sensor. Reproduced with permission.^[^ ^15^ ^]^ Copyright 2021, Springer Nature Limited. C) CRISPR microneedles platform.

With the COVID‐19 pandemic, several research groups have applied wearable sensors to monitor the health of infected patients remotely.^[^ ^173^, ^175^, ^176^ ^]^ However, wireless sensing technology of wearable sensors is affected by physical distance, humidity, temperature of the surroundings, and signal intensity.^[^ ^173^ ^]^ In addition, ideal wearable materials must exhibit several characteristics including flexibility, stretchability, transparency, adhesion properties, water repellency, selective permeability, and biocompatibility.^[^ ^177^ ^]^ Wearable devices are desirable for accurate and portable diagnosis, particularly those integrated with CRISPR‐based technologies, microfluidic devices, and materials science.^[^ ^123^ ^]^ Combined with synthetic biology, a wearable device was designed to detect SARS‐CoV‐2, which included origami‐based sample collection and reaction components including sampling, RT‐RPA amplification, and SHERLOCK reaction units (**Figure** **9**A).^[^ ^15^ ^]^ With the downstream LFA signaling, it achieved the detection limit of 17 aM within 90 min. Wearable molecular diagnosis was achieved by integrating the sensor and freeze‐dried, cell‐free (wFDCF) reaction into a facemask. The orthogonal Cas12a sensor in the reaction layers accepts the targets from the collection layers by capillary action, resulting in fluorescence or colorimetric readouts through the detector mounted on the smartphone or wireless signal transmission (Figure 9B). Similarly, a wearable collector was designed to concentrate SARS‐CoV‐2 RNA and was integrated into commercial masks (N95, KN95, surgical, and textile masks).^[^ ^37^ ^]^ This microfluidic device increases the convenience of sampling and virus testing, which contributes to preventing and controlling airborne diseases.

A CRISPR microneedle (MN) platform was designed to bridge the gaps in real‐time in vivo monitoring and amplification‐free detection.^[^ ^130^, ^171^ ^]^ As shown in Figure 9C, electrically sensitive graphene was integrated into an MN patch, in which the extraction and real‐time monitoring of Epstein‐Barr virus (EBV) cfDNA in interstitial fluid (ISF) proceeded in a minimally invasive manner.^[^ ^178^ ^]^ After carboxyl graphene was dropped on the MN, its terminal carboxyl groups were covalently bound to dCas9, resulting in the automatic conjunction of ribonucleoproteins and biomarkers in the ISF.^[^ ^179^ ^]^ The three‐electrode MN system was modified and subsequently applied by in vivo differential pulse voltammetry testing. This wearable CRISPR‐based MN device detected EBV cfDNA with fM sensitivity in 30 min, and long‐term in vivo screening was achieved.^[^ ^130^ ^]^

## 5 Challenges and Future Perspectives

The limitations of current clinical diagnostic techniques are a major challenge for the effective diagnosis of emerging infectious diseases, which accelerates innovation in next‐generation CRISPR‐based microfluidic biosensors with superior performance characteristics, including 1) low cost, 2) high sensitivity and specificity, 3) field deployability, and 4) multiplexed adaptability. However, challenges that limit the mature implementation and possible commercialization of CRISPR‐based microfluidic systems remain vital bottlenecks in effective infectious diseases diagnosis, urgently requiring continuous improvement.

### 5.1 Ultrasensitive Detection

Sensitivity is critical in infectious diseases diagnosis, particularly for specimens with low pathogen concentrations. Therefore, the ideal CRISPR‐based microfluidic system requires easy fabrication, operation workflow, and ultrasensitive detection. Because amplification and labeling strategies can increase assay time and cost, new materials or structures could be utilized as sensor elements to further enhance the sensitivity of the CRISPR‐based microfluidic system. Although several strategies, such as amplification‐free strategies with multiple on‐surface electronic or digital microdevices, have been exploited to substantially enhance the detection sensitivity of CRISPR‐based biosensing, the reliable detection of low‐concentration analytes remains a major challenge because of nonspecific background signals. To address this challenge, integrated microfluidic systems with analyte concentrations using specific bioreceptors (such as molecularly imprinted polymers and aptamers), portable digital microfluidic devices, or sensitive signal output modes (gFETs and optofluidic biosensors) are potential solutions for ultrasensitive CRISPR/Cas diagnosis of infectious diseases.^[^ ^180^ ^]^

### 5.2 Multiplexed and High‐Throughput Screening

Microfluidics is becoming increasingly appealing for high‐throughput screening owing to recent trends in the prevention of infectious diseases, necessitating testing for large sample sizes and various analytes. However, there are still gaps in conjunction with multiple functionalities and compatibility with large‐scale integration of multiple assays, which fundamentally limits the application of CRISPR‐based microfluidic systems in multiplexed and high‐throughput screening. Recently, isothermal NASBA sequencing‐based high‐throughput test (INSIGHT) has achieved population‐scale testing by combining NGS.^[^ ^181^ ^]^ Therefore, the NGS‐based multiplexed CRISPR/Cas system with near‐patient diagnosis may be feasible for regular population‐scale screening in future pandemics. Furthermore, the inclusion of automatic robotic techniques in the CRISPR‐based microfluidic system may be another solution for high‐throughput detection, which can keep track of various pathogens from a large sample size.^[^ ^182^ ^]^ In the future, these novel technologies will be promising approaches for combining CRISPR‐based microfluidics as a revolutionary biosensing strategy in molecular diagnosis.

### 5.3 Self‐Contained and Portable Platforms

Traditional microfluidic technologies tend to perform sophisticated processes in multiple steps, resulting in reagent leakage and unwanted contamination during the manipulation. In addition, sampling and transportation of the active virus before molecular diagnosis raise biosafety concerns.^[^ ^183^ ^]^ Therefore, microfluidic devices should also consider these factors so that precautions can be taken to eliminate these adverse effects on the detection performance. Accordingly, a self‐contained and portable CRISPR‐based microfluidic diagnosis with an automated all‐in‐one system that can integrate sampling, CRISPR/Cas reactions, and signaling is vital for field‐deployable infectious diseases diagnosis, particularly in low‐resource settings. For example, the self‐powered integrated microfluidic point‐of‐care low‐cost enabling (SIMPLE) may be an alternative development tendency, which integrates a pre‐patterned amplification initiator, automated plasma separation device, and self‐powered microfluidic pumping in a fully portable chip, enabling autonomous on‐chip detections without external processing.^[^ ^159^ ^]^ A handheld imaging system equipped with portable CRISPR‐based microfluidic biosensing can realize POCT with low contamination risks during high‐throughput nucleic acid detection. Additionally, low‐cost, self‐driven paper‐based microfluidics with portable readers and automated wearable devices will contribute to a self‐contained and portable CRISPR/Cas biosensing platform for effective diagnosis.^[^ ^64^, ^184^ ^]^ The COVID‐19 pandemic has imposed instant requirements for rapid and accurate commercial screening of SARS‐CoV‐2 and the SHERLOCK and DETECTR‐associated SARS‐CoV‐2 detection kits have been approved by the U.S. Food and Drug Administration (FDA) as excellent CRISPR microfluidics in nucleic acid biosensing.

### 5.4 Real‐Time Monitoring

The miniaturized, highly biocompatible wearable medical devices for in situ and in vivo monitoring of pathogens can realize real‐time response to the infection status, which has drawn increasing attention.^[^ ^185^ ^]^ Combining CRISPR‐based microfluidic biosensing with wearable devices can meet the needs of many diseases that require the detection of certain pathogens for a period of time. However, the potential instability and restricted sensitivity during real‐time monitoring are central barriers to further commercialization of CRISPR‐based microfluidic diagnosis, which should be actively developed.^[^ ^186^ ^]^ Additionally, the wireless interface for CRISPR‐based microfluidic diagnosis should be improved so that robust battery‐free operation and simultaneous data acquisition are available.^[^ ^187^ ^]^ Wearable CRISPR microfluidic devices have emerged with excellent capability for real‐time monitoring of a large influx of modern personalized medicine with flexibility and integration capabilities. With the upcoming Internet of Things, future decentralized CRISPR‐based microfluidic diagnosis will enable patient‐centralized information collection and artificial intelligence‐driven models for real‐time diagnosis and precision treatment of infectious diseases.^[^ ^188^, ^189^ ^]^

### 5.5 Disease Prediction

The global COVID‐19 pandemic has exposed the weakness in the prediction system for emerging infectious diseases, particularly for undiagnosed pathogens.^[^ ^190^ ^]^ Delayed diagnosis leads to hysteretic treatment, poorer prognosis, and antibiotic abuse.^[^ ^191^ ^]^ Even though the advantages of CRISPR‐based microfluidic diagnosis are superior, the prediction of novel pathogen outbreaks is still beyond its capability. Because CRISPR‐based microfluidic systems can provide portable and automated detection, this detection strategy is a good candidate for rapid pathogen prediction in the future. Typically, the use of NGS, bioinformatics, and artificial intelligence may overcome the drawbacks of CRISPR‐based microfluidic diagnostic methods for their application in infectious diseases prediction.^[^ ^192^ ^]^

**Figure 10:**
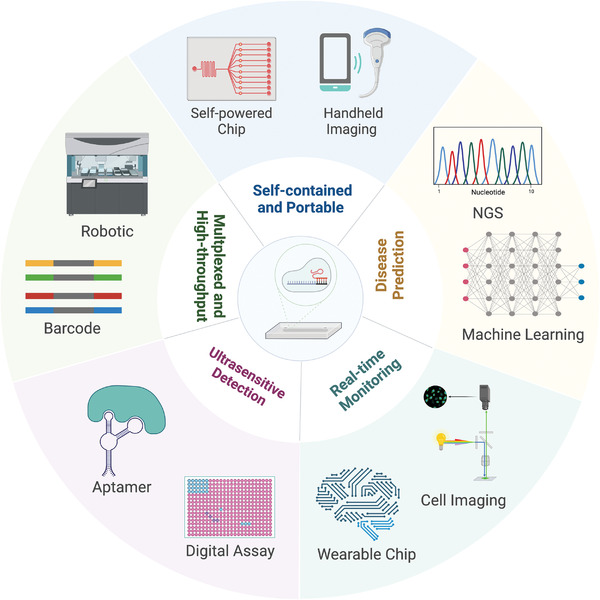
Future perspectives in CRISPR‐based microfluidic technologies.

In conclusion, future CRISPR‐based microfluidics will likely intersect with materials science,^[^ ^37^ ^]^ electromagnetism,^[^ ^57^, ^113^ ^]^ acousto‐optics, synthetic biology,^[^ ^15^ ^]^ and bioinformatics to detect pathogens in a highly integrated microfluidic chip (**Figure** **10**). In addition, ultrasensitive CRISPR‐based diagnosis combined with affordable and portable microfluidic devices may facilitate real‐time monitoring, extensive data analysis, and precision medicine in future patient‐centric automatic medical care. Clinical trials are required to verify the novel CRISPR‐based microfluidic diagnosis to ensure its validity after clinical implementation.

## 6 Conclusions

Current studies on CRISPR‐based microfluidic platforms have described their potential to develop convenient, practical, and portable diagnoses of infectious diseases, particularly in the field of POCT. As summarized above, various CRISPR‐based microfluidic diagnosis methods have been successfully developed based on state‐of‐the‐art strategies, such as paper, digital, and integrated wearable platforms. With the outstanding advantages of integrated, automatic, sensitive, and specific properties, we believe that CRISPR‐based microfluidic diagnosis is promising for use in next‐generation molecular diagnosis. Combined with portable signal readouts, real‐time monitoring, and high‐throughput capabilities, the CRISPR‐based microfluidic system represents a revolutionary breakthrough in the practical diagnosis of infectious diseases.

## Conflict of Interest

The authors declare no conflict of interest.
